# A case report: Feasibility of a near infrared ray vision system (Photo dynamic eye®) for the postoperative ischemic complication of gallbladder carcinoma

**DOI:** 10.1016/j.ijscr.2018.11.007

**Published:** 2018-11-13

**Authors:** Koichiro Sakata, Daiki Kijima, Takashi Furuhashi, Katsuhiko Morita, Toshihiko Abe

**Affiliations:** a Japan Seafares Relief Association Moji Ekisaikai Hospital, Japan; b JCHO Shimonoseki Medical Center, Japan

**Keywords:** GB ca, gallbladder carcinoma, GB, gallbladder, CT, multi-detector computed tomography, MR, diffusion weighted magnetic resonance, PET, positron emission tomography, Gallbladder carcinoma, Near-infrared ray vision system, Preservation of the bile duct, Ischemic complication, Bile leakage, Bile duct stricture

## Abstract

**Introduction:**

Radical resection with or without preserving extra-hepatic bile duct has shown similar prognoses for gallbladder carcinoma (GB ca), although aggressive resection is essential. In preserving bile duct, ischemic complications are serious, life-threatening serious problems. Correct evaluation of the blood flow to the biliary tract is crucial.

**Case presentation:**

A case of gallbladder ca in a 62-year-old man was reported. The patient was diagnosed with stage Ⅱ GB ca (T2, N0, M0) by ultrasonography, computed tomography, magnetic resonance imaging, and endoscopic ultrasonography. Cholecystectomy and intraoperative frozen section examination were planned. After recognition of invasion to subserosa and negative cystic duct margin, radical dissection of the lymph nodes in the hepatoduodenal ligament with preserving biliary tract was performed. Three days after the operation, biliary peritonitis was revealed. Emergency laparotomy showed ischemic bile leakage. Proper blood flow of the biliary system was realized by a near-infrared ray vision system (Photo Dynamic Eye®: HAMAMATSU Photonics) using indocyanine green. Primary suture of the extra-hepatic biliary duct and T-tube drainage were selected. No stricture of the bile system nor recurrence was recognized for two years after surgery.

**Discussion:**

In the case of ischemic biliary complications, whether to preserve the extrahepatic bile duct is a critical issue for the surgeons. In this case study, the feasibility of authentic indocyanine green near-infrared imaging was shown for postoperative ischemic biliary situations.

**Conclusions:**

Authentic indocyanine green near-infrared imaging was feasible for the estimation of the blood flow to the postoperative ischemic biliary complication.

## Highlights

• Radical resection with or without preserving extra-hepatic bile duct has shown similar prognoses for gallbladder carcinoma.
• In the case of ischemic biliary complications of gallbladder carcinoma, whether to preserve the extrahepatic bile duct is a critical issue.
• Authentic indocyanine green near-infrared imaging is feasible for the estimation of the correct blood flow in postoperative ischemic biliary complications.

## 1 Introduction

The value of cholecystectomy with radical resection for gallbladder carcinoma (GB ca) remains debatable, although aggressive surgery is important in improving the long-term prognosis for GB ca, for which surgical treatment results are dismal and prognoses poor [[1], [2], [3], [4]]. In preserving bile duct, ischemic complications are serious, life-threatening serious problems. Correct evaluation of the blood flow to the biliary tract is crucial.

## 2 Case presentation

**Fig. 1:**
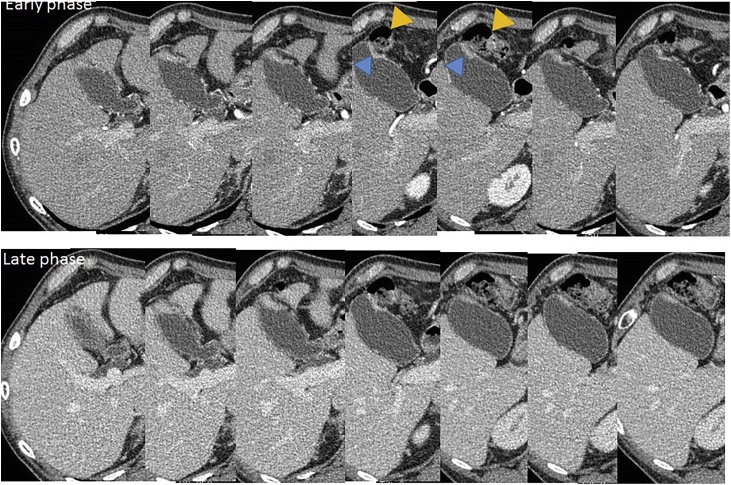
Computed Tomography Images. (For interpretation of the references to colour in this figure legend, the reader is referred to the web version of this article.) Enhanced mass was shown at the tail of the gallbladder (blue triangle). Tumor was adjacent to the transverse colon (yellow triangle).

**Fig. 2:**
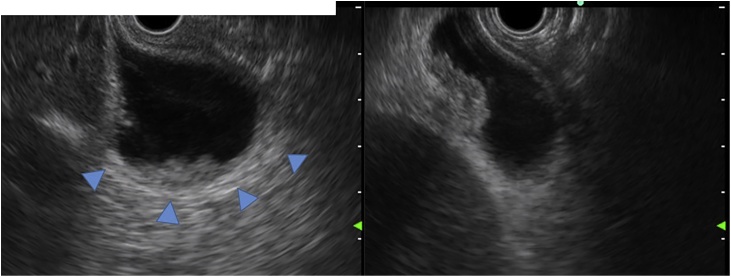
Endoscopic Ultrasonography Imaging. (For interpretation of the references to colour in this figure legend, the reader is referred to the web version of this article.) The continuity of the 3^rd^ layer was confirmed (blue triangle). The invasion depth was assumed to be subserosa.

**Fig. 3:**
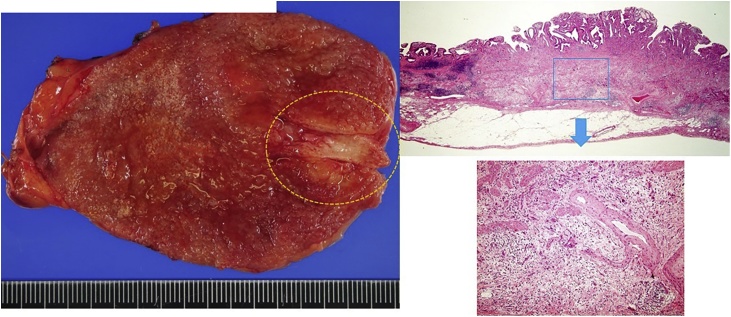
Pathological Examination. Poorly differentiated adenocarcinoma was shown. According to the classification of biliary tract cancers established by the Japanese Society of Hepato-Biliary-Pancreatic Surgery (3rd English edition), S0; Hinf1; H0; Binf0; PV0; A0; P0; N0; M(-); ST(-); pT2; pStageⅡ; BM0; HM0; EM0; CurA, were diagnosed.

**Fig. 4:**
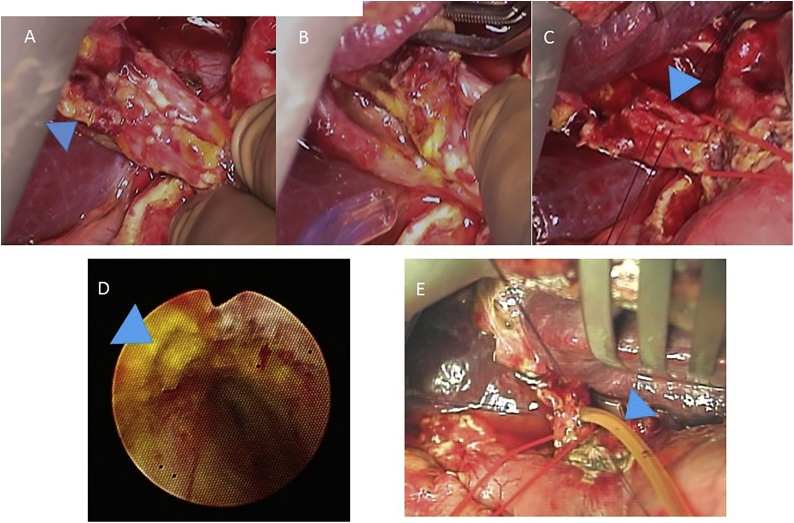
Findings at the Emergency Intraoperative. (For interpretation of the references to colour in this figure legend, the reader is referred to the web version of this article.) Emergency laparotomy revealed the ischemic bile leakage at the connecting points of the hepatic, cystic, and common bile duct (A, B): discoloring of the cystic duct. Ulceration and perforation in the root of the cystic duct was detected by intraoperative cholangioscopy after choledocotomy (C, D). T-tube drainage was performed (E).

**Fig. 5:**
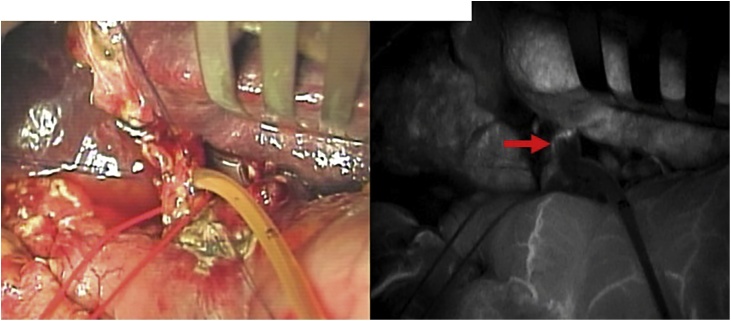
Indocyanine Green Near-Infrared Image. (For interpretation of the references to colour in this figure legend, the reader is referred to the web version of this article.) Indocyanine Green Near-Infrared Image showed the flow of arterial network on the common bile duct (red arrow).

A case of gallbladder carcinoma was reported in a 62-year-old man, with whom tumor in the gallbladder (GB) was occasionally detected without symptom. He had suffered from alcoholic hepatitis and diabetes mellitus at 50 years. There were no special notes in his family history, or in his relevant physical examination and other significant clinical findings. At admission, carcinoembryonic antigen level was 2.3 ng/ml and carbohydrate antigen 19-9 level was 8.4 U/ml. Ultra-sonographic images showed the low echoic mass in the fundus of the GB without any signal of blood flow (Fig. 1). Multi-detector computed tomography (CT) images showed the mass adjacent to the transverse colon without lymph-node enlargement. Diffusion weighted magnetic resonance (MR) images showed no deformity of the GB and no lymph-node swelling. Endoscopic ultrasonography revealed the continuity of the 3^rd^ layer of the gallbladder wall: invasion to the subserosa layer (Fig. 2). Positron emission tomography (PET) showed the low-grade accumulation at the tumor in SUV max 2.5 at early phase and late phase. The patient was diagnosed with GB ca at the stage Ⅱ: T2, N0, M0, according to the classification of biliary tract cancers established by the Japanese Society of Hepato-Biliary-Pancreatic Surgery (3rd English edition). Cholecystectomy and intraoperative frozen section examination were planned. After the recognition of the invasion depth to subserosa and negative cystic duct margin, lymph-node dissection of the hepatoduodenal ligament with preserving biliary tract was performed. The blood flow of the common bile duct was estimated as remaining intact macroscopically. Pathological examination revealed the same progression stage as proposed preoperatively (Fig. 3). Three days after operation, biliary peritonitis was diagnosed. Emergency laparotomy revealed ischemic bile duct leakage at the connecting points of the hepatic, cystic, and common bile duct; discoloration of the cystic duct; and ulceration and perforation at the root of the cystic duct (Fig. 4). A near-infrared ray vision system (Photo Dynamic Eye®) using indocyanine green was introduced to estimate the blood flow. After the recognition of the proper flow (Fig. 5), preservation of the extrahepatic biliary duct was selected. No stricture of the bile system nor recurrence was recognized for two years after surgery.

## 3 Discussion

Radical resection with or without preserving the extra-hepatic bile duct showed similar prognoses for GB ca in stages I-III [5], although preoperative diagnoses of the extension into the subserosa or further invasion into the hepatoduodenal ligament were difficult [6]. Recently, diagnostic precision has been improved by endoscopic ultrasonography, which revealed the accuracy for the clinical staging of GB ca [[7], [8], [9]], and by CT and MR images for diagnosis of metastatic lymph nodes in biliary carcinomas [10]. In preserving bile duct, ischemic complications of the bile duct, such as bile leakage, bile duct stricture, etc., are serious life-threatening problems. In the case of ischemic biliary complications, whether to preserve the extrahepatic bile duct is a critical issue for the surgeons. Thus, the correct evaluation of the blood flow is essential. Indocyanine green near-infrared imaging offers a promising way to assess perfusion at the site intended for anastomosis [11,12]. In this case study, the feasibility of an authentic indocyanine green near-infrared ray vision system (Photo Dynamic Eye®: HAMAMATSU Photonics) was shown for postoperative ischemic biliary trouble.

## 4 Conclusions

Authentic indocyanine green near-infrared imaging using Photo Dynamic Eye® is feasible for the estimation of the blood flow in postoperative ischemic biliary complications.

## Conflicts of interest

We have nothing to declare.

## Funding

This research did not receive any specific grant from funding agencies in the public, commercial, or not-for-profit sectors.

## Ethical approval

This case report was approve by the ethics committee in Japan Seafares Relief Association Ekisaikai Moji Hospital.

## Consent

Authors obtained written and signed consent to publish a case report from the patient.

## Authors' contributions

Koichiro Sakata: study concept or design

Daiki Kijima: data collection, data analysis or interpretation

Koichiro Sakata, Toshihiko Abe, Takashi Furuhashi, Katsuhiko Morita: writing the paper

## Registration of research studies

This is a case report.

## Guarantor

None.

## Provenance and peer review

Not commissioned, externally peer reviewed.

Copyright transfer agreement: All authors agree with copyright transfer.
